# Relationships between horizontal jump kinematics and sprint performance in female sprinters and team sport athletes

**DOI:** 10.3389/fspor.2025.1640223

**Published:** 2025-08-20

**Authors:** Bjørn Johansen, Roland van den Tillaar, Jonathon Neville

**Affiliations:** ^1^ Department of Sports Sciences, Nord University, Levanger, Norway; ^2^ Sports Performance Research Institute New Zealand, AUT University, Auckland, New Zealand

**Keywords:** sprint performance, horizontal jumps, single leg jump, bounding, contact time, kinematics, female athletes

## Abstract

The main objective of this study was to investigate how different horizontal jump exercises relate to sprint performance in female athletes, and whether these relationships differ between sprinters and team sport athletes. Twelve female sprinters (age 18.9 ± 3.7 yrs) and twelve team sport athletes (football/handball; age 16.5 ± 2.5 yrs) performed 40 m sprints along with four 30 m horizontal jump tests comprised of: bounding and single leg jumps, each performed for either speed or distance. For single leg jumps, both legs were tested, and the best result was used for analysis. Kinematic variables—horizontal velocity, step length, contact time, flight time, and step frequency—were analyzed across all tasks. A two-way mixed-design ANOVA revealed significant main effects of test type and group, and significant interactions for all variables (*p* < 0.05). Sprinters showed higher horizontal velocity, longer step length, and shorter contact times across most sprint and jump conditions. Horizontal velocity in the single leg jump for speed showed the strongest correlations with sprint velocity across both groups, with particularly strong associations in sprinters (*r* = 0.70–0.92). Bounding for speed also correlated strongly with sprint performance in the team sport group (*r* = 0.57–0.68), but less so in sprinters. Sprint contact time and step length showed variable but often strong associations with corresponding parameters in the jump tests, particularly in the single leg jump for speed. These findings suggest that selected horizontal jump tests may be effective tools for both performance assessment and sprint-specific training.

## Introduction

In many sports, great demands are placed on some form of speed or sprinting ability (1). While both acceleration and maximal velocity are important for sprint events in track and field (2), the ability to accelerate quickly over the first 10–20 m is particularly important in game-based sports such as football and handball, where repeated short bursts of speed are required. This has been emphasized in several articles highlighting the biomechanical demands of early acceleration and its importance for sports performance (3–5). To develop sprinting velocity, in addition to sprinting itself, a wide range of exercises can be used, such as resisted and assisted sprints, as well as horizontal plyometric exercises including bounding and single leg jumps (2, 4, 6, 7).

**Figure 1:**
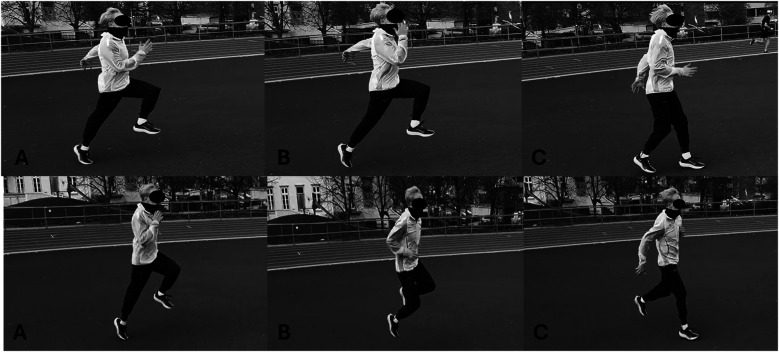
Movement sequences in two horizontal jump tests. Top: bounding. Bottom: single leg jump. Each exercise is shown in three phases: **(A)** take-off, **(B)** midair, and **(C)** landing.

Sprint running and horizontal jumps share key biomechanical features, including rapid force production, effective use of the stretch-shortening cycle, and alternating phases of ground contact and flight (8). In the early acceleration phase (0–10 m), the velocity is relatively low (6–7 m/s), ground contact times are longer (≈0.13–0.15 s), and step frequency increases rapidly before stabilizing around 4.5–5.0 Hz (6, 9). Step length increases from ∼1.2 m in the first steps to over 2 m by 20 m (10), where most trained sprinters reach 80%–85% of their maximal velocity (11). At maximal velocity, elite sprinters typically exceed 10 m/s, with contact times around 0.10–0.12 s, and step lengths above 2 m. Faster athletes generally exhibit longer steps, shorter ground contact times, and higher step frequency, while flight time remains relatively stable across performance levels (9, 12).

Horizontal jump exercises differ in their movement characteristics. Bounding for speed typically involves high step frequency (∼3–4 Hz), moderate step lengths (∼2 m), short contact times, and horizontal velocities around 7–8 m/s (6, 13). Bounding for distance emphasizes maximal displacement per step, with longer contact and flight times (∼0.2–0.4 s) and lower velocity (<6 m/s) (2, 14). Single leg jumps for distance feature low frequency (<2 Hz), long steps (∼2.5–3.0 m), and extended contact phases (15). In contrast to distance-based jumps, single leg jumps for speed are performed over a fixed distance (e.g., 30 m) with the sole aim of maximizing horizontal velocity, rather than achieving the longest possible jump with each step. Although the movement pattern differs from sprinting, the shared performance objective may provide insight into forward-oriented force production, particularly in the early acceleration phase (6, 15).

Numerous studies have examined the relationship between jumping ability and sprint performance. Both acceleration and maximal velocity have been linked to performance in horizontal and vertical jump tests. Several studies have shown strong associations, particularly for maximal velocity (16, 17), but also for acceleration phases (7, 18). Explosive strength and short ground contact times have been highlighted as key predictors of sprint performance, especially during early acceleration (19). A recent meta-analysis by Lin et al. (20) found inverse correlations ranging from moderate (*r* ≈ 0.45–.48) for single-jump tests to very large (up to *r* = 0.76) for multiple-jump tests, especially over acceleration distances. However, most of these studies have relied on outcome measures such as jump height or distance, offering little insight into the step-by-step mechanics of movement (18, 20). Furthermore, they have primarily used bilateral or vertical jump formats—such as countermovement or squat jumps—while more sprint-specific horizontal movements like bounding for speed have received comparatively little attention. Although the majority of studies have overlooked sprint-specific jump formats, a few have highlighted their relevance. For example, Washif and Kok (14) found a strong correlation between 10 speed-bounds and maximal sprint speed in young male sprinters. Similarly, McCurdy et al. (21) reported that unilateral jumps were more strongly associated with sprint performance than bilateral jumps in female soccer athletes. These findings point to the potential value of horizontal and unilateral jump tests—yet such approaches remain rare in the literature.

To our knowledge, no studies have investigated single leg jumping for speed over extended distances (e.g., 30 m) as a predictor of sprint performance. While unilateral jump tests have been linked to sprint ability—mainly via vertical or distance-based formats—horizontal single leg speed jumps remain unexplored in this context (15, 22). This is especially relevant since such tests emphasize repeated unilateral force application with the single goal of forward velocity— closely resembling sprinting during acceleration due to its unilateral and forward-oriented force application (6, 15). Bounding for speed, although biomechanically similar to sprinting in step frequency, contact time, and rhythm, is technically demanding and places conflicting demands on frequency and step length. In contrast, we speculate that single leg jumping for speed may involve lower absolute velocity but a more natural, self-organizing forward movement pattern, potentially offering higher specificity for sprint acceleration.

Most studies in this field have focused on homogeneous athlete samples, either sprinters or team sport athletes. By comparing two distinct groups—female sprinters and team sport athletes—our study allows for an investigation of whether group differences influence movement patterns and the strength of correlations across sprint and jump parameters. This approach may provide new insight into how training background and motor strategies affect the expression of horizontal force and velocity.

The aim of this study was to investigate and compare sprint and horizontal jump kinematics—specifically horizontal velocity, step length, contact time, flight time, and step frequency—in female sprinters and team sport athletes. We hypothesized that the speed-oriented jump exercises, due to their kinematic similarity to sprinting, would show the strongest correlations with sprint performance. In particular, we expected single leg jump for speed to demonstrate stronger associations with sprint velocity than bounding for speed, given that it emphasizes forward propulsion without additional technical constraints. We also hypothesized that sprinters would exhibit higher horizontal velocity, longer step length, and shorter contact times than team sport athletes across most test conditions.

## Materials and methods

### Participants

Twenty-four female athletes participated in the study, divided into two groups: twelve experienced local female sprinters (age 18.9 ± 3.8 years, body mass 60.9 ± 5.8 kg, body height 1.71 ± 0.05 m) with personal best 100 m times of 13.10 ± 0.6 s and twelve experienced female team sports athletes (handball and football) (age 16.5 ± 2.6 years, body mass 61.2 ± 4.1 kg, body height 1.67 ± 0.04 m) from local clubs participated in this study. All had ≥6 years of continuous training in their sport and competed at regional or national junior level. Sprinters were on average older than team sport athletes (18.9 ± 4.0 vs. 16.5 ± 2.5 years), although the difference was not statistically significant (*p* = 0.086). This age difference may still reflect greater training exposure or specialization in the sprint group and should be considered when interpreting group differences. An *a priori* power analysis was conducted using G*Power version 3.1 (23). Based on recommendations by Cohen (24), a medium effect size (*f* = 0.25) was selected as a realistic and conservative estimate for between-group differences in biomechanical variables in sports performance studies. With *α* = 0.05 and power set at 0.80, the analysis indicated that a total sample size of 24 participants would be sufficient to detect statistically significant effects. Participants were thoroughly informed about the procedures, potential risks, and benefits of the study, both in written and verbal formats. Written consent was collected before any testing, and parental consent was obtained for all participants. The study complied with the latest revision of the Declaration of Helsinki and was approved by the Norwegian Agency for Shared Services in Education and Research (project number 957478).

### Procedure

Before testing, all participants completed their usual warm-up routines. For sprinters, this typically included sprint drills and light plyometric exercises, while team sport athletes followed the type of warm-up they normally used before regular football or handball training. This was followed by a short familiarization period on the horizontal jumps, performing 1–2 practice attempts of each jump test. The test leader provided instructions and brief feedback to ensure proper execution and consistent performance. The participants then performed two maximum attempts in running shoes on a tartan track in an indoor athletics hall. Recovery periods between each attempt were 2–3 min, which is considered sufficient to allow near-complete neuromuscular recovery during maximal sprint and plyometric efforts (25). The sprint distance was 40 m, while all jump tests were performed over 30 m. In all tests, participants started from a stationary position without any external signal. In sprint and bounding, they began from a standing position with a self-selected lead foot; in single leg jumps, they started standing on the tested leg. The sprint test was always performed first, followed by the six horizontal jump tests in randomized order: single leg jump (SLJ) for distance (left and right), SLJ for speed (left and right), bounding for distance, and bounding for speed (Figure 1).

### Measurements

Contact and flight times during sprints were measured using inertial measurement units (IMUs), while an infrared optical contact mat (Ergotest Technology AS, Langesund, Norway) was used for jumps. The IMUs (Ergotest Technology AS, Langesund, Norway) featured a wireless 3-axis accelerometer (±16 g, accuracy ±1.0%), gyroscope (2,000 deg/s, accuracy ±1.0%), and magnetometer (±1,300/2,500 µT, accuracy ±5%), with a sampling frequency of 200 Hz and a mass of 20 grams. They were securely attached to the dorsal side of each foot using tape and contact and flight times during sprints were calculated by analyzing the velocity patterns associated with plantar flexion and extension of both feet (23, 26). For jumps, the infrared optical contact mat consists of two units, each 2.5 cm thick and 0.87 m long and operate at a sampling frequency of 1,000 Hz. These were placed at the beginning and end of the 20-meter testing zone. Together, they generate a layer of infrared light approximately 5 mm above the surface. When this light is interrupted by foot contact with the ground, the system registers these events to determine contact and flight times. Horizontal displacement for each participant was tracked using a laser device (Noptel Oy, Oulu, Finland) aimed at the lower back throughout the test. This device employs a CMP3 distance sensor (Noptel Oy, Oulu, Finland) to continuously measure distance over time, with a sampling rate of 2.56 kHz. Based on these measurements, horizontal velocity was calculated from the distance covered during ground contact as recorded by the laser. All sensor recordings were synchronized and processed using Musclelab software version 10.200.90.5095 (Ergotest Technology AS, Langesund, Norway).

Horizontal velocity, step length, contact time, flight time, and step frequency were measured during both sprints and horizontal jumps. For each condition, average values from consecutive steps were selected to best represent performance. For maximal sprint velocity and the jump-for-speed exercises, the 3–4 steps with the highest horizontal velocity were used. For single leg jumps, both legs were tested, and the best trial—defined as the highest horizontal velocity for speed conditions and the longest distance for distance conditions—was used for analysis, regardless of leg. For the jump-for-distance exercises, the steps with the greatest horizontal displacement were selected. For the 10 m and 20 m sprint segments, horizontal velocity was obtained exactly at the distance marker, and step variables were taken from the nearest step. If the midpoint between two steps was closer to the marker, the average of those two steps was used.

In both sprinting and horizontal jump conditions, step length (m) was defined as the horizontal distance between two consecutive ground contacts. Contact time and flight time were defined as the durations of ground contact and airborne phase, respectively, while step frequency (Hz) was calculated as the inverse of total step time, and horizontal velocity (m/s) was calculated from displacement over time during ground contact.

### Statistics

Before conducting the main analyses, the normality of each variable was assessed using the Shapiro–Wilk test. A two-way mixed-design ANOVA was conducted separately for each dependent variable: horizontal velocity, step length, contact time, flight time and step frequency. The within-subjects factor was test type (seven levels: 10 m sprint, 20 m sprint, maximal sprint, bounding for speed, bounding for distance, single leg jump for speed, and single leg jump for distance), and the between-subjects factor was training group (sprinters vs. team sport athletes). Sphericity was assessed using Mauchly's test, and Greenhouse–Geisser corrections were applied when the assumption was violated. Significant main effects and interactions were followed up with pairwise comparisons using the Least Significant Difference (LSD) method and estimated marginal means. Effect sizes were reported as partial eta squared (*η*^2^).

Pearson's correlation coefficients (*r*) were then calculated to examine the relationships between sprint performance (velocity at 10 m, 20 m, and maximal velocity) and selected kinematic variables from the jump tests (velocity, contact time, and step length). No correction for multiple comparisons was applied to the correlation analyses, as these were exploratory and based on specific *a priori* hypotheses grounded in the existing literature. Given the importance of high horizontal velocity in sprinting, and the need to identify which jump-related kinematic factors best explain variation in this capacity, horizontal sprint velocity was chosen as the primary outcome variable in the correlation analyses. As a key indicator of sprint performance, velocity is strongly influenced by both step length and contact time. Correlation coefficients were interpreted using standard thresholds: *r* = ±0.10–0.29 (weak), ±0.30–0.49 (moderate), ±0.50–0.69 (strong), and ±0.70–1.00 (very strong), as recommended by Bhandari (27). All statistical analyses were performed using JASP version 0.18.3.0, with the level of significance set at *p* < 0.05.

### Results

**Figure 2:**
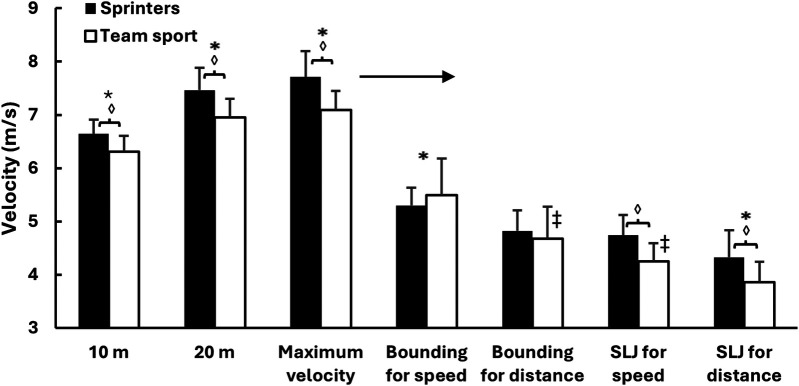
Mean ± SD horizontal velocity across sprint distances and horizontal jump conditions in sprinters and team sport athletes. * Indicates a significant difference with all other exercises for both groups on a *p* ≤ 0.05 level. ‡ Indicates a significant difference with all other exercises for this group on a *p* ≤ 0.05 level. ◊ Indicates a significant difference between these two groups for this condition on a *p* ≤ 0.05 level. → Indicates a significant difference between sprint parameters and jumps for both groups on a *p* ≤ 0.05 level.

**Figure 3:**
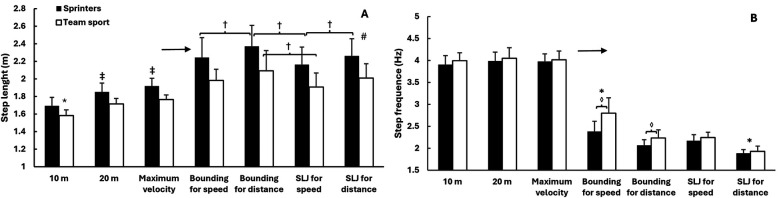
Mean ± SD on step length **(A)** and frequency **(B)** across sprint distances and horizontal jump conditions in sprinters and team sport athletes. * Indicates a significant difference with all other exercises for both groups on a *p* ≤ 0.05 level. → Indicates a significant difference between sprint parameters and jumps for both groups on a *p* ≤ 0.05 level. ‡ Indicates a significant difference with all other exercises for this group on a *p* ≤ 0.05 level. † Indicates a significant difference between these two conditions for this group on a *p* ≤ 0.05 level. # indicates a significant difference between the groups for all conditions on a *p* ≤ 0.05 level. ◊ Indicates significant difference between groups for this condition on a *p* ≤ 0.05 level.

**Figure 4:**
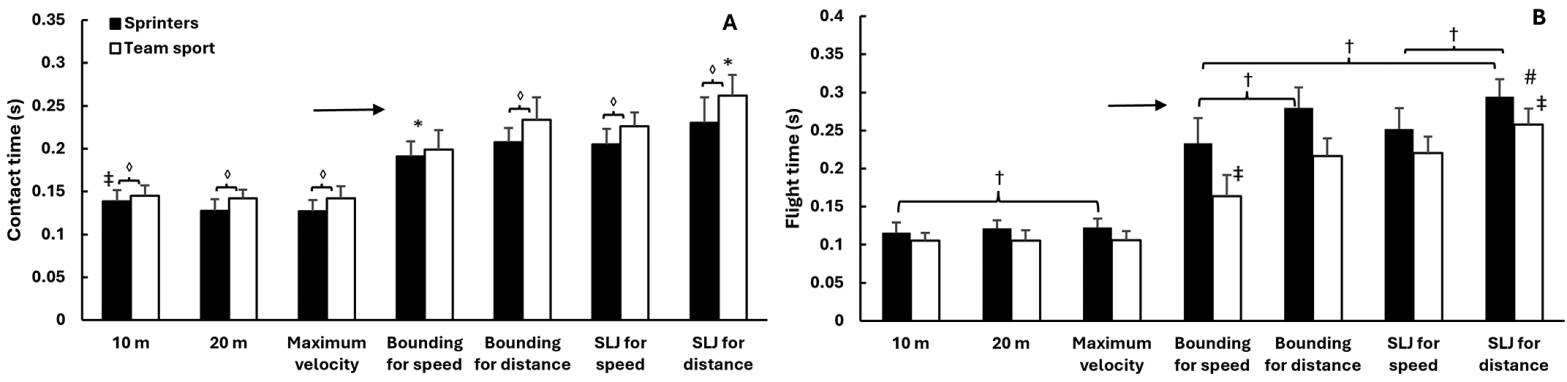
Mean ± SD contact time **(A)** and flight time **(B)** across sprint distances and horizontal jump conditions in sprinters and team sport athletes. * Indicates a significant difference with all other exercises for both groups on a *p* ≤ 0.05 level. → Indicates a significant difference between sprint parameters and jumps for both groups on a *p* ≤ 0.05 level. ‡ Indicates a significant difference with all other exercises for this group on a *p* ≤ 0.05 level. † Indicates a significant difference between these two conditions for this group on a *p* ≤ 0.05 level. ◊ indicates a significant difference between the two groups for this condition on a *p* ≤ 0.05 level. # indicates a significant difference between the two groups for all conditions on a *p* ≤ 0.05 level.

A two-way mixed ANOVA revealed significant main effects of test type for all five kinematic variables (*F* ≥ 87.4, *p* < 0.001, *η*^2^ ≥ 0.799), reflecting substantial differences across sprint and jump conditions. Significant group differences were also observed (*p* ≤ 0.039, *η*^2^ ≥ 0.180), and test × group interactions were found for four of the five variables (*p* ≤ 0.041, *η*^2^ ≥ 0.124). *Post hoc* comparisons revealed that sprinters reached significantly higher velocities than team sport athletes in all sprint conditions (10 m, 20 m, and maximal; all *p* < 0.001). In the jump conditions, sprinters showed significantly higher velocities in bounding for distance (*p* = 0.005), single leg jump for speed (*p* = 0.004), and single leg jump for distance (*p* < 0.001), while team sport athletes had higher velocity in bounding for speed (*p* = 0.016). Contact time was significantly shorter for sprinters in all sprint conditions and in both single leg jump types (all *p* < 0.001). Flight time was significantly longer for sprinters in bounding for distance, single leg jump for speed, and single leg jump for distance (*p* ≤ 0.004), while no group differences were observed in the sprint conditions or in bounding for speed. Step length was greater for sprinters in bounding for distance and single leg jump for distance (both *p* < 0.001), but did not differ significantly in the other jumps. For step frequency, significant group differences were observed only in bounding for speed and bounding for distance, where team sport athletes showed higher frequencies (*p* < 0.05) (Figures 2–4).

Several significant correlations were found between sprint performance and horizontal jump parameters (Table 1). Among sprinters, the strongest relationship was observed between sprint velocity and velocity in the single leg jump for speed (*r* = 0.83–0.92). Sprint velocity also correlated strongly with step length in both single leg jump types (*r* = 0.64–0.71) and in bounding for distance (*r* = 0.59–0.69). Contact time in sprinting was strongly associated with contact time in single leg jump for speed (*r* = –0.65 to −0.69) and in bounding for speed (*r* = –0.73 to −0.74). For team sport athletes, sprint velocity showed strong correlations with velocity in bounding for speed (*r* = 0.57–0.68), and moderate to very strong correlations with single leg jump for speed (*r* = 0.50–0.76), especially at 20 m and maximal velocity. Step length during sprinting was most strongly related to step length in bounding for distance (*r* = 0.37–0.59), while the associations with single leg jump for distance were weaker and less consistent (*r* = –0.09 to 0.58). Sprint contact time also correlated strongly with contact time in single leg jump for speed (*r* = 0.59–0.67), but only weakly to moderately with bounding for speed (*r* = 0.23–0.36).

**Table 1:** Pearson's correlation coefficients (*r*) between sprint parameters and kinematic variables from horizontal jump tests in sprinters and team sport athletes.

Sprint parameter	Jump variable	10 m		20 m		Max velocities	
		Sprint	Team sport	Sprint	Team sport	Sprint	Team sport
Sprint velocity	Bounding for speed velocity	0.39	0.66*	0.46	0.68*	0.46	0.57*
	Single leg jump for speed velocity	0.92*	0.50	0.83*	0.76*	0.87*	0.70*
	Bounding for speed step length	0.43	0.48	0.52	0.14	0.58*	0.15
	Bounding for distance step length	0.59*	0.51	0.65*	0.31	0.69*	0.19
	Single leg jump for speed step length	0.71*	0.68*	0.64*	0.75*	0.71*	0.63*
	Single leg jump for distance step length	0.70*	0.32	0.61*	0.33	0.67*	0.34
	Bounding for speed contact time	−0.21	- 0.56	−0.18	−0.79*	−0.17	−0.76*
	Bounding for distance contact time	−0.45	−0.11	−0.40	−0.35	−0.42	−0.35
	Single leg jump for speed contact time	−0.70*	−0.28	−0.68*	−0.57	−0.65*	−0.58*
	Single leg jump for distance contact time	−0.59*	- 0.11	−0.53	−0.45	−0.50	−0.54
Step length	Bounding for distance step length	−0.02	0.37	0.49	0.59*	0.70*	0.46
	Single leg jump distance step length	0.27	−0.09	0.41	0.58	0.55	0.21
Contact time	Bounding for speed contact time	0.60*	0.36	0.82*	0.23	0.74*	0.32
	Single leg jump speed contact time	0.47	0.67*	0.74*	0.59*	0.69*	0.67*

*Indicate a significant correlation on *p* < 0.05.

## Discussion

The aim of this study was to compare sprint and horizontal jump kinematics between sprinters and team sport athletes, and to examine how jump kinematics are related to sprint kinematics. We hypothesized that the speed-oriented jump exercises, particularly the single leg jump for speed, would show the strongest correlations due to their biomechanical similarity to sprinting. We also expected sprinters to exhibit higher velocity, longer step length, and shorter contact time across most conditions.

One of the main findings in this study was that sprinters and team sport athletes differed systematically across most sprint and jump conditions. In line with our hypotheses, sprinters showed higher horizontal velocity, longer step length, and shorter contact time—both during sprinting and in the jump exercises. These differences are consistent with previous findings indicating that sprint-specific training improves horizontal force production and reactive strength (9, 10, 13).

One of the key findings in this study was a strong to very strong correlation between velocity in the single leg jump for speed and sprint velocity in both sprinters (*r* = 0.83–0.92) and team sport athletes (*r* = 0.50–0.76). Although performed at substantially lower absolute velocity—approximately 60% of maximal velocity—this exercise appears to closely reflect the intent of sprinting—rapid forward movement achieved through short ground contacts and high horizontal force output, without the added technical focus on jump distance (1, 8). The strongest associations were found at the velocity measured at 10 m and 20 m in the sprint, where athletes rely on high levels of horizontal force production and relatively long ground contact time (1, 8). These characteristics also define the single leg jump for speed, which may help explain its strong relationship with sprint velocity and its value as a sprint-specific assessment, particularly during early acceleration (11, 15). Similar findings have been reported by Maulder and Cronin (7), who found that horizontal single leg jump performance was predictive of sprint acceleration, highlighting the importance of unilateral concentric force production in early sprint phases. In addition to the strong correlations, sprinters showed significantly higher velocity and shorter contact time in this test, further supporting its relevance for identifying sprint-specific qualities.

Bounding for speed showed contrasting relationships with sprint performance across the two groups. In team sport athletes, the correlation with sprint velocity was strong (*r* = 0.57–0.68), while in sprinters it was only moderate (*r* = 0.39–0.46). This partly contradicts our hypothesis, which assumed that bounding for speed would consistently correlate strongly with sprinting due to its horizontal orientation and rhythmic similarity. One likely explanation is differences in movement execution, as bounding for speed combines high step frequency with long steps—an inherently technical combination that sprinters likely perform more consistently due to experience. Among team sport athletes, however, several participants appeared to perform the movement in a more running-like pattern rather than true bounding, as observed during testing. This may have increased both velocity and resemblance to sprinting in that group and could explain why team athletes achieved higher relative velocity and frequency in bounding for speed. Despite the weaker correlation among sprinters, the movement pattern in bounding for speed—with short contact times and high step frequency—closely resembles sprinting mechanics, particularly during the transition from acceleration to top velocity (6). This resemblance supports its continued use in sprint training (14). and aligns with previous findings showing that bounding-type exercises are among the most sprint-specific jump tests, with strong correlations to sprint performance across various athlete populations (28).

The jump-for-distance tests—bounding for distance and single leg jump for distance—were performed at considerably lower velocity than sprinting and involved longer contact and flight times, reflecting their emphasis on horizontal displacement rather than speed. Despite these biomechanical differences, velocity in sprinters correlated strongly with step length in both jumps, especially at maximal velocity (*r* up to 0.71), suggesting that such tasks can still reflect sprint-relevant capacities like horizontal power and leg extension mechanics (11, 20). In contrast, correlations between jump step length and sprint step length were more variable, indicating individual differences in movement transfer. For the team sport athletes, distance-based jumps showed weaker and less consistent associations. This may reflect a lower level of technical familiarity with horizontal jumps aimed at maximizing distance, as such movements are rarely emphasized in football and handball training. Bounding for distance correlated more clearly with sprint step length than with sprint velocity, whereas single leg jump for distance showed the widest variability, possibly due to limited specificity or technical inconsistency (18, 21). Correlations—particularly those involving step length—tended to increase with sprint distance, especially in sprinters, aligning with the growing importance of step length at higher velocities (10, 29) and suggesting that jump-derived metrics may be more informative when analyzing top-speed sprint mechanics.

Beyond velocity measures, contact time in the jumps was also strongly associated with sprint contact time. Among sprinters, both bounding for speed and single leg jump for speed showed strong correlations, suggesting that athletes who minimize ground contact during sprinting tend to do the same in these jump tests (r up to 0.83). This supports the idea that these exercises capture neuromuscular qualities such as reactive strength and rapid force production (1, 8). In the team sport group, single leg jump for speed showed moderate to strong associations with sprint contact time, while bounding for speed was more weakly related—possibly reflecting differences in movement control or technical familiarity. Similar patterns have been observed in previous studies, where horizontal jump tests, especially those emphasizing speed, were found to relate closely to sprint contact dynamics in team sport athletes (7, 28). These findings support the idea that such tests can offer valuable insight into individual sprint mechanics, particularly contact dynamics.

The findings of this study suggest that the single leg jump for speed may be particularly valuable for assessing sprint-specific characteristics in both sprinters and team sport athletes. It showed strong associations with sprint velocity and contact time, especially in the acceleration phase, and demonstrated consistent performance differences between the two groups. These results, along with the exercise's directional similarity to sprinting, highlight its potential as a sprint-specific assessment tool based on its clear relationships with relevant performance variables. Bounding for speed also showed relevant associations, particularly in the team sport group, but with greater variability. This may reflect technical inconsistencies, as the exercise requires combining high frequency and long steps—demands that sprinters typically execute more fluently due to training background. Overall, the study supports the use of sprint-specific horizontal jumps as informative tools for performance assessment in both training and talent identification contexts.

### Limitations and future directions

Several limitations should be considered when interpreting the results. The relatively small sample size (*n* = 12 per group) limits the generalizability of the findings and may increase the influence of individual variation. Although the exclusive inclusion of female athletes allowed for direct group comparisons, the results may not extend to male populations. There was also a small age difference between groups, with sprinters being slightly older than the team sport athletes (*p* = 0.086). This may reflect greater training exposure or specialization in the sprint group and could have contributed to some of the performance differences observed. While a brief familiarization period was included before testing, it is possible that limited experience with certain jump tests, particularly among some team sport athletes may still have influenced execution and performance. Finally, the step parameters at 10 m and 20 m were derived from one or two steps near fixed distance markers, rather than full segment-level analyses, which may introduce minor variability.

Future studies should examine whether similar patterns are observed in male athletes and in larger, more heterogeneous samples. Longitudinal research evaluating the effects of targeted jump training—especially single leg jump for speed—would also help determine whether improvements in specific jump tasks translate to enhanced sprint performance. Further exploration of how motor learning and exercise familiarity influence jump execution would also be valuable in applied testing environments.

## Conclusion

This study demonstrated that the single leg jump for speed is a strong indicator of sprint performance in both sprinters and team sport athletes. It showed the highest correlations with sprint velocity and contact time, particularly during the acceleration phase, and clear performance differences between groups. Bounding for speed also showed relevant associations, especially in the team sport group, but with greater variability. Jump-for-distance tests were less biomechanically similar to sprinting, yet still reflected certain sprint characteristics—primarily step length—especially among sprinters. Overall, the findings support the use of sprint-specific horizontal jumps as complementary tools for assessing sprint capacity and movement mechanics in female athletes.

## Data Availability

The original contributions presented in the study are included in the article/Supplementary Material, further inquiries can be directed to the corresponding author.
